# Laparoscopic Drainage of Severe Intra-abdominal Abscesses Following Cesarean Section: A Case Report

**DOI:** 10.7759/cureus.83616

**Published:** 2025-05-06

**Authors:** Kazunori Masahata, Takuya Kushimoto, Shohei Maekawa, Rikuto Hirose, Kenshi Wasada

**Affiliations:** 1 Pediatric Surgery, Aizenbashi Hospital, Osaka, JPN; 2 Obstetrics and Gynecology, Aizenbashi Hospital, Osaka, JPN

**Keywords:** abscess, cesarean section, drainage, laparoscopic surgery, peritonitis

## Abstract

We report a rare case of a 29-year-old pregnant woman with obesity who developed a severe intra-abdominal abscess after an emergency cesarean section (CS) for labor arrest. The patient presented with persistent fever and lower abdominal pain after CS. *Enterococcus faecalis* was isolated from the purulent material in the pelvis. Despite antibiotic treatment, the patient’s symptoms and laboratory test results did not improve. Abdominal CT revealed multiple intra-abdominal abscesses and distended bowel loops. On the 12th day of admission, laparoscopic drainage was performed under general anesthesia because image-guided drainage using interventional radiology was not feasible. Laparoscopic adhesiolysis and peritoneal lavage were performed, and drainage tubes were placed. The patient’s condition gradually improved; she was discharged without complications. Intra-abdominal abscess formation after CS is a rare but potentially fatal complication. Although no standard treatment has been established, laparoscopic drainage may be an effective alternative, particularly when conservative management fails and the percutaneous drainage of abscesses is unsuitable.

## Introduction

Intra-abdominal abscess formation after a cesarean section (CS) is rare because of the widespread use of antibiotic prophylaxis. The most common sites are the leaves of the broad ligament, the pouch of Douglas, and the anterior uterus [1]. Thus far, no standard treatment has been established for this condition, and its management remains challenging. Conservative management with antibiotics is often the first-line treatment for patients with surgical site infections after CS. However, these infections are often resistant to antimicrobial therapy, and image-guided drainage is the standard intervention [2,3]. This approach may be challenging for patients with multiple intra-abdominal abscesses. Exploratory laparotomy drainage is the treatment of choice when surgical intervention is required. Laparoscopic surgery is rarely performed in patients with pelvic or intra-abdominal abscesses after CS, owing to limited visualization of the enlarged uterus and inflammatory adhesions. Despite these challenges, a recent study showed that laparoscopic surgery for pelvic abscesses is a viable alternative that reduces the risk of postoperative adhesions, postoperative pain, and length of hospitalization compared with the use of laparotomy [4,5].

Herein, we report a rare case of acute intra-abdominal abscess formation extending from the pelvis to the peritoneal cavity after CS that was successfully treated with laparoscopic surgery. This case highlights the feasibility of laparoscopic surgery for selecting patients for whom conservative and image-guided approaches are not viable options.

## Case presentation

**Figure 1:**
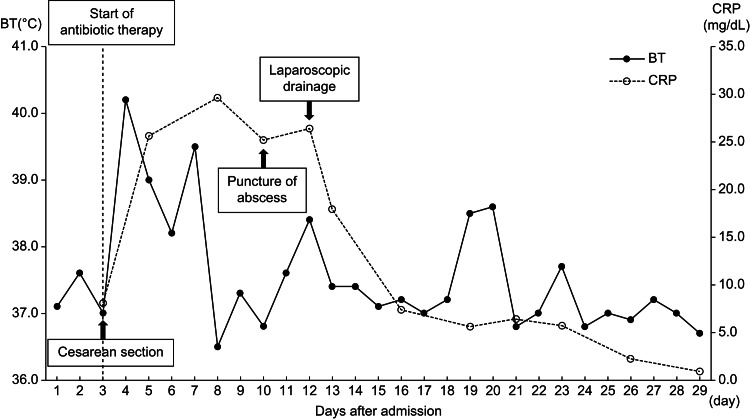
Clinical course of the patient BT, body temperature

A 29-year-old obese woman (gravida 1, para 0) with gestational diabetes was admitted to our hospital for labor induction at term. On the third day of admission, the patient developed a high fever, and laboratory tests revealed elevated WBC and CRP levels. The clinical diagnosis was chorioamnionitis; antibiotic therapy with IV ampicillin (8 g/day) and gentamicin (480 mg/day) was initiated. The patient underwent emergency CS for labor arrest at 40 weeks and one day of gestation. The standard surgical procedure was performed using the classical Pfannenstiel method. The intraoperative bleeding volume was approximately 930 ml, including the amniotic fluid, and CS was completed without complications. The clinical course of the patient is shown in Figure 1.

The patient complained of persistent fever and lower abdominal pain after CS. The culture of the placental swab at CS revealed *Enterococcus faecalis*. We performed antimicrobial susceptibility testing and evaluated the minimum inhibitory concentration (MIC) of various antibiotic agents. In accordance with these results, antibiotic therapy was switched to a combination of IV ampicillin (8 g/day), gentamicin (480 mg/day), and clindamycin (2.4 g/day); this combination was administered for 10 days. Despite antibiotic treatment, the patient’s symptoms persisted, and the laboratory test results did not improve. Abdominal CT revealed a 50 × 16 mm inhomogeneous fluid collection located anterior to the uterus without abscess formation; the small bowel loops were distended and fluid-filled.

**Figure 2:**
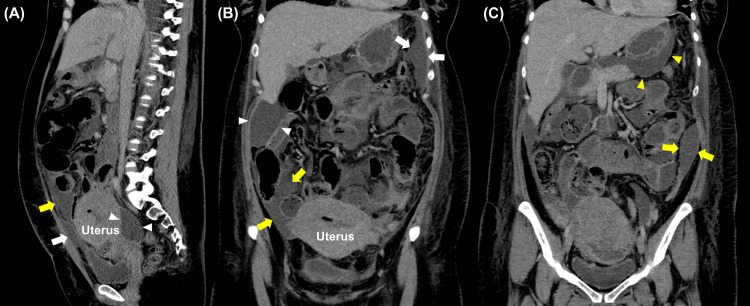
Contrast-enhanced abdominal CT image showing intra-abdominal abscesses extending into the peritoneal space The sagittal image (A) shows abscesses anterior to the uterus (white arrow), beneath the anterior abdominal wall (yellow arrow), and in the pouch of Douglas (white arrowheads). Coronal images (B, C) show abscesses in the left subphrenic space (white arrows), paracolic gutters (yellow arrows), hepatic flexure (white arrowheads), and omental bursa (yellow arrowheads).

Samples collected during ultrasound-guided fluid aspiration revealed the presence of *E. faecalis*. The lowest MIC was observed for ampicillin (2 µg/ml) and penicillin G (2 µg/ml). Despite continued antibiotic administration, the patient’s symptoms worsened, and blood tests revealed elevated CRP levels. On the 12th day after admission, contrast-enhanced abdominal CT revealed a 110 × 20 mm inhomogeneous collection of fluid extending from the pelvis into the abdominal cavity; this was highly suggestive of intra-abdominal abscesses (Figure 2).

Although image-guided drainage was considered, it could not be performed because of the complicated abscesses extending into the peritoneal cavity and distended bowel loops. Although surgical drainage of the abscesses, which were widespread in the abdominal and pelvic cavities, was required, the patient was obese (BMI: 32 kg/m²). Exploratory laparotomy was considered a high-risk procedure because a large incision would be required. Therefore, the patient underwent laparoscopic drainage under general anesthesia in the lithotomy position. Multiport laparoscopic drainage was then performed. After a 3-cm vertical skin incision was made at the umbilicus, a multiple-instrument single-access port (Applied Medical, Rancho Santa Margarita, USA) was first inserted into the peritoneal cavity, followed by the placement of 12-mm and 5-mm assistant ports in the gel port and carbon dioxide insufflation at 10 mmHg. Then, the laparoscope was inserted into the abdomen using an advanced access platform.

**Figure 3:**
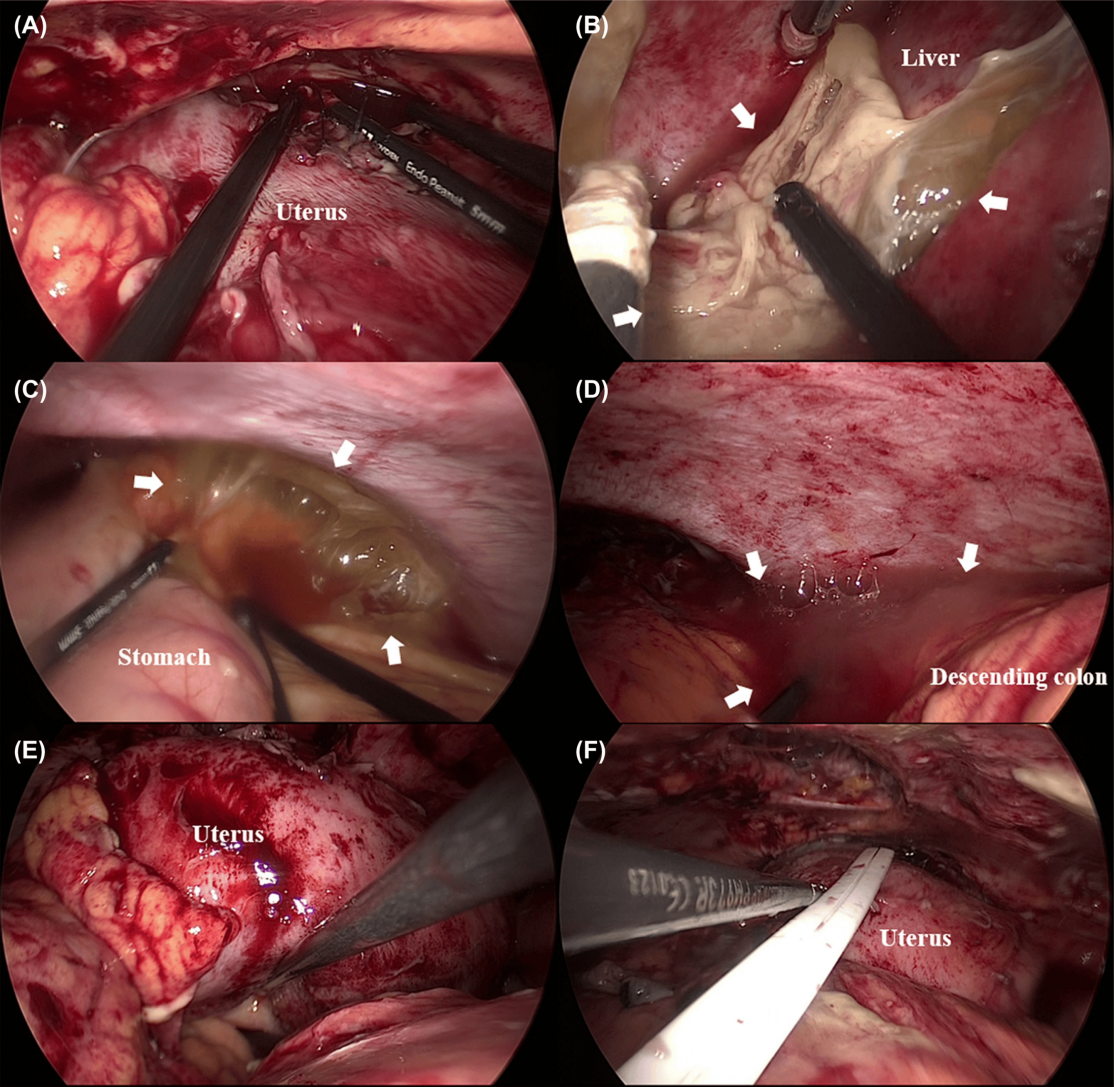
Intraoperative findings Laparoscopic adhesiolysis and peritoneal lavage were performed for intra-abdominal abscesses involving the area anterior to the uterus (A), hepatic flexure (B), left subphrenic space (C), left paracolic gutter (D), and the pouch of Douglas (E). The white arrows indicate the abscess cavity. Panel F shows the drainage tube placement in the abscess cavity (F).

Upon entering the abdominal cavity, abscess adhesions containing yellowish pus were observed, covering the omentum and abdominal wall, particularly in the pelvic cavity (Figure 3).

After general inspection, adhesions were carefully dissected from the abdominal wall using a single-port umbilical approach. Subsequently, two working ports were placed under direct visualization of the scope at the level of the umbilicus on the left and right flanks of the abdomen. A large amount of purulent and turbid fluid was noted in the peritoneal cavity, with extensive fibrinous exudates over the liver, bowel loops, omental bursa, and pelvic cavity. Laparoscopic adhesiolysis and lavage were also performed. Then, the peritoneal fluid was aspirated, and fibrinous exudate was removed to the greatest degree possible. Next, a peritoneal fluid culture was performed. We used a uterine manipulator during laparoscopic surgery, as the visualization of the pelvic space was hampered because of the uterus enlargement after CS and the formation of inflammatory adhesions. The abscesses were completely opened and washed with saline.

At the end of the laparoscopy, two drainage tubes were placed in the abscess cavity. Intraoperative complications were not observed. Peritoneal fluid culture did not show any bacterial growth. Gram staining of the peritoneal fluid revealed the presence of polymorphonuclear leukocytes but no pathogens. We suspected *Mycoplasma hominis*, which can cause pelvic abscesses. Antibiotic therapy was switched to a combination of IV minocycline (200 mg/day) and sulbactam/ampicillin (12 g/day). Although the patient complained of persistent fever and lower abdominal pain postoperatively, the symptoms had ameliorated, and the results of the blood tests gradually improved after the continuous administration of antibiotics. The drainage tubes were removed on postoperative day 5. The patient was discharged on postoperative day 17.

## Discussion

In the current report, we present a rare case of rapid abscess formation after widespread abscesses in the peritoneal cavity despite antibiotic prophylaxis. CS increases the risk of obstetric complications and is associated with hemorrhagic complications, surgical site infections, pelvic abscesses, and endometritis [6]. Diffuse abscess formation in the intra-abdominal cavity is a rare complication of postpartum endometritis, occurring in less than 2% of patients [1]. Tissue adhesion in the pelvic cavity adversely affects fertility, and diffuse peritonitis caused by pelvic abscess rupture can lead to potentially fatal sepsis [7]. Therefore, intra-abdominal abscesses after CS should be promptly and effectively treated to prevent complications.

Although abscess formation after CS is classically treated with antibiotics, thus far, no standard treatment has been established for this condition. However, the success rate of IV antibiotics alone in patients with pelvic abscesses is approximately 70% [8]. If the abscess is localized, image-guided drainage is a viable option because it is less invasive than other methods. The success rate of image-guided drainage for pelvic abscesses is approximately 90% [9,10]; however, abscess sites can cause limitations. In the present case, image-guided drainage was difficult because of complicated intra-abdominal abscesses that extended into the peritoneal space and distended bowel loops. Traditionally, exploratory laparotomy has been used to treat acute peritonitis and abscesses.

Laparoscopy is contraindicated because of poor visualization, difficult trocar access due to distended bowel loops, residual abscess formation, peritoneal adhesions, and the possibility of iatrogenic intestinal injury. The conversion rate of laparoscopic surgery in cases of acute peritonitis is up to 23.3%, most frequently because of peritoneal adhesions [11]. However, the laparoscopic approach has been gradually accepted owing to advancements in surgeons’ technical skills and the advantages of small wounds, faster recovery, less intraoperative blood loss, shorter length of hospitalization, and fewer complications [12-14]. To the best of our knowledge, thus far, there have only been a few reported cases of pelvic abscesses being treated laparoscopically after CS in the literature [15].

As observed in the present case, the laparoscopic approach has several advantages over laparotomy for managing complications after CS. Laparotomy results in a larger incision being made because of multiple abscesses extending into the pelvic and abdominal cavities and obesity. Laparoscopic surgery is a minimally invasive approach that involves a small incision, less damage to the abdominal wall, faster recovery, and a lower risk of bowel obstruction. A good surgical field is achievable during laparoscopy with port selection, which considers an enlarged uterus. Laparoscopy has a magnifying effect, clearly displays the surgical area, and can be used to observe the target lesions from multiple angles. Therefore, the laparoscopic approach provides better-quality peritoneal lavage in diffuse abdominal areas, such as the pouch of Douglas, subphrenic space, and paracolic gutters.

Additionally, the abdomen was not accessed through the previous surgical scar, which enabled better visualization. In the present case, we used a multiple-instrument single-access port (Applied Medical), which was first inserted into the peritoneal cavity, followed by the insertion of 12-mm and 5-mm assistant ports into the gel port. The multiple-instrument single-access port has been widely used for single-site laparoscopic surgery because it can be used through small incisions ranging from 1.5 to 3 cm [16,17]. The Alexis wound protector/retractor of the multiple-instrument single-access port offers atraumatic retraction and a flexible fulcrum for easy instrument movement during laparoscopic surgery. In the present case, adhesions were carefully dissected from the abdominal wall through the umbilical single-port site using an open technique. The multiple-instrument single-access port is useful for dissecting adhesions during laparoscopic surgery in patients with severe intra-abdominal abscesses. Uterine manipulators are widely used in laparoscopic surgery because uterine mobilization is essential for exposing the pelvic space and increasing the surgeon’s field of vision [18]. In the present case, the uterine manipulator served as a useful surgical tool for dissecting pelvic adhesions during laparoscopic drainage, which is particularly important for ameliorating severe pelvic abscess formation and uterus enlargement after CS.

## Conclusions

This case report demonstrates that laparoscopic drainage is a feasible and effective approach for managing multiple intra-abdominal abscesses after CS, particularly in cases of antibiotic therapy resistance and in those for which image-guided drainage is not feasible. These findings provide critical insights into the role of minimally invasive surgery in post-CS complications and suggest that laparoscopy may be a valuable alternative to laparotomy in selected patients.

Although this report focused on a single case, further research is required to evaluate the long-term outcomes, recurrence rates, and broader applicability of laparoscopic drainage in a larger patient cohort. By addressing these challenges, this report will establish a foundation for expanding the use of laparoscopic surgery in the management of complex post-CS infections, thereby improving patient outcomes and recovery times.
